# Bilateral Transcranial Magnetic Stimulation of the Prefrontal Cortex Reduces Cocaine Intake: A Pilot Study

**DOI:** 10.3389/fpsyt.2016.00133

**Published:** 2016-08-08

**Authors:** Corinna Bolloni, Riccardo Panella, Mariano Pedetti, Anna Grazia Frascella, Cristiana Gambelunghe, Tommaso Piccoli, Giuseppe Maniaci, Anna Brancato, Carla Cannizzaro, Marco Diana

**Affiliations:** ^1^Department of Experimental Biomedicine and Clinical Neuroscience, University of Palermo, Palermo, Italy; ^2^Laboratory of Cognitive Neuroscience ‘G. Minardi’, University of Sassari, Sassari, Italy; ^3^Ser.T, ‘Health’s House n.1’, A.S.L. No. 1 Umbria, Perugia, Italy; ^4^Legal Medicine, Forensic Science and Sports Medicine Section, Department of Surgical and Biomedical Science, University of Perugia, Perugia, Italy; ^5^Department of Health Promotion and Maternal Care ‘G. D’Alessandro’ University of Palermo, Palermo, Italy; ^6^Laboratory of Cognitive Neuroscience ‘G. Minardi’, Department of Chemistry and Pharmacy, University of Sassari, Sassari, Italy

**Keywords:** cocaine use disorder, dopamine, PFC, rTMS

## Abstract

**Background:**

Chronic cocaine consumption is associated with a decrease in mesolimbic dopamine transmission that maintains drug intake. transcranial magnetic stimulation (TMS) is gaining reliability, a useful therapeutic tool in drug addiction, since it can modulate cortico-limbic activity resulting in reduction of drug craving.

**Aims:**

In the present study, we investigated the therapeutic effect of bilateral TMS of prefrontal cortex (PFC) in reducing cocaine intake, in a sample of treatment-seeking patients with current cocaine use disorder (DSM-V).

**Methods:**

Ten cocaine addicts (DSM-V) were randomly assigned to the active or sham stimulation protocol in a double-blind experimental design. Twelve repetitive TMS (rTMS) sessions were administered three times a week for 4 weeks at 100% of motor threshold, over bilateral PFC. Cocaine intake (ng/mg) was assessed by hair analysis at baseline (before treatment, T0), after 1 month (end of treatment, T1), 3 (T2), and 6 (T3) months later. All subjects received psychological support weekly.

**Results:**

The two-way ANOVA for repeated measures did not show a significant effect of the interaction between time and treatment (*F*_4,32_ = 0.35; *p* = 0.87). Despite that result indicated no difference in the effect of the two conditions (active vs. sham) along time, a decreasing trend in cocaine consumption in active TMS group (*F*_3,23_ = 3.42; *p* = 0.04) vs. sham (*F*_3,15_ = 1.88; *p* = 0.20) was observed when we performed exploratory analysis with time as factor. Indeed, *Post hoc* comparisons showed a significant reduction in the amount of cocaine detected from the onset to 3 months later (T0–T2; *p* = 0.02) and to the end of treatment (T0–T3; *p* = 0.01) in addicts from the active group.

**Conclusion:**

Bilateral rTMS of PFC at 10 Hz did not show a significant effect on cocaine intake compared to sham. However, a long-term reduction on cocaine intake in active TMS-treated patients was observed when we considered the time as factor. Further studies are required to confirm these encouraging but preliminary findings, in order to consolidate rTMS as a valid tool to treat cocaine addiction.

## Introduction

Substance use disorders (SUD) represent a major public health concern in the western world, with about 27.7 million young adult users in the last year (1). A general consensus has emerged on drug addiction as a substance-induced, aberrant form of neural plasticity (2–5). At the neurotransmitter level, dopamine (DA) plays a key role in the neurobiological mechanisms underlying cocaine use disorders (CUD) (6, 7). Indeed, preclinical studies in rodents have shown a profound reduction in DA-ergic activity in cocaine-withdrawn rats and a resulting decrease of DA release in the nucleus accumbens (Nacc) (8). In parallel, human imaging studies have shown a reduction in DA D2 receptors in the ventral striatum of detoxified cocaine-dependent subjects (6) and a blunted DA release as indexed by a reduction of amphetamine-induced DA release in the limbic striatum (7).

These observations are the fundamental building blocks of the DA hypothesis of drug addiction (2), which ascribes to the hypo-functioning DA system, a key role in drug abuse and leads to theorize that functional “boosting” of the DA signaling (9) may have beneficial effects in reducing drug intake.

In spite of recent significant progress in understanding the neurobiology of SUD, therapeutic advances have proceeded at a slower pace (10, 11), in particular for CUD.

Among the various interventions available for addiction, transcranial magnetic stimulation (TMS) (12) represents a non-pharmacological tool and a testable opportunity in the treatment of CUD (13, 14) given its capability to target and modulate specific brain circuits. Previous studies on the therapeutic efficacy of TMS on drug addiction have focused on the evaluation of craving and drug intake in nicotine-dependent subjects (15–17), alcoholics (18, 19), and cocaine addicts (20–22). Despite the variability of the stimulation protocols applied, the clinical studies highlighted a significant reduction in different measures of addictive behavior. Unfortunately, the relatively small number of studies, substantial inter-study heterogeneity, and lack of head-to-head comparison studies make it difficult to identify common factors associated with beneficial outcomes. Moreover, discrepancies in the geometry of the magnetic field generated by standard figure-of-eight coil and therefore in the brain area targeted by the stimulation, may account for the transient effects observed in previous studies (22, 23), Recently, a novel probe called H-coil has shown more consistent and prolonged effects, presumably due to its ability to stimulate the subcortical regions of the brain that are involved in the neuropathology of addiction (23–27). Specifically, the H-coil can stimulate fronto-striatal circuits that govern cognitive control through the efferent projections from pyramidal neurons in the PFC to the DA-containing midbrain neurons and other subcortical areas. Given these previous findings, we sought to investigate the effect of bilateral rTMS of PFC on cocaine intake in treatment-seeking subjects affected by CUD and diagnosed according to DSM-V criteria (28).

## Materials and Methods

The study was conducted at the “National Health Service for Addiction” of the “Health’s House 1” in Marsciano (A.S.L. No 1 Umbria, Perugia, Italy). All the procedures were conformed to the Good Clinical Practice. The Ethics Committee’s approval was received on 16th December 2010. All subjects gave written informed consent in accordance with the Declaration of Helsinki (Ethics Committee’s Registration n. 1695/10).

### Participants

Patient enrollment was conducted from November 2011 to November 2014. All the subjects were seeking outpatient treatment for CUD according to DSM-V criteria (28). The inclusion criteria were: age between 18 and 65 years; an average cocaine consumption during the 4 weeks before screening of 2 days/week for a total minimum consumption ranging from 0.5 to 20 ng/mg of cocaine amount at the baseline; able to understand and sign the informed consent; being motivated to stop the intake. Exclusion criteria were: concurrent SUD (except for tobacco smoking); lifetime history or a co-morbidity of psychiatric or neurological disorders; chronic medical illness; current use of psychotropic medications; medical devices (pace-maker, metal implants, device for inflating); family history of epilepsy; pregnancy; involvement in other clinical trial; or in a legal action (29, 30). All patients signed their written, informed consent.

### Experimental Procedure

All patients underwent toxicological assessments before treatment (T0), 4 weeks later (end of treatment-T1), and after 3 and 6 months (follow-up at T2 and T3). We used the hair test as indirect measure of drug intake because it provides long-term information about drug consumption with higher sensitivity and specificity than urine analysis (31–33). Through the analysis, we monitored the amount of cocaine, cannabis, and opioids over the course of the experiment.

#### Hair Analysis

##### Reagents and Chemicals

Solvents were from Merck (Milano, Italy). *N*-methyl-*N*-trimethylsilyltrifluoroacetamide (MSTFA) was obtained from Sigma-Aldrich (Milano, Italy). Cocaine, benzoylecgonine (BEG), and their deuterated analogs were obtained from Chemical Research 2000 (Rome, Italy).

##### Procedure

A 50 mg hair sample was taken close to the scalp near the *posterior vertex*. All hair samples were analyzed using a fully validated method (34). Briefly, the hair samples were washed, cut accurately, dried, and spiked with deuterated internal standards. The samples were hydrolyzed with 1 ml of 0.1M HCl overnight at 40°C. After liquid/liquid extraction (2 × 5 ml chloroform/hexane/isopropanol 50:17:33 v/v/v at pH 8.0) and derivatization with 50 ml of MSTFA (70°C, 30 min), 1 μl of the aliquot was submitted to gas chromatography/mass spectrometer (Thermo Fisher Scientific, Milano, Italy) analysis in selected ion monitoring mode. Ion monitoring parameters were: cocaine (82, 182, 303), cocaine D3 (85, 185, 306), BEG TMS (82, 240, 361), and BEG D3 TMS (85, 243, 364). The target ions used for quantification are underlined. Standard calibration curves were prepared from blank hair fortified with standard solutions. Calibration lines were checked for linearity using the least squares regression method. Adequate linearity was observed for all compounds with a correlation coefficient (*R*^2^) above 0.99. Calculated limits of detection (LOD, S/N ≥3) and limits of quantification (LOQ, S/N ≥5) were 0.01 and of 0.02 ng/mg, respectively, for each compound. As recommended by Society of Hair Testing guidelines (35), we applied the 0.50 ng/mg cutoff for cocaine and the 0.05 ng/mg cutoff for BEG. Precision (expressed as percent variation coefficient, CV%) and accuracy (expressed as bias%) were assessed by intraday and interday analyses and were less than 20%.

### TMS Device

We used the H1-coil TMS device (Brainsway Ltd., Jerusalem, Israel) (23, 36). This technology is composed by a power generator equipped with a cooling system connected by a mechanic arm to a plastic helmet in which is embedded the coil. This device is able to produce both sham and active stimulation by using a magnetic card linked to a magnetic reader situated near the touch screen monitor where the parameters of stimulation can be set. The activation of the sham coil mimics the acoustic sounds of the “active” one without inducing magnetic fields or discomfort for the subject. In this way, each patient received the stimulation protocol based on the randomized card assigned (37). During the stimulation session, the subject was instructed to sit below the helmet, which was located by the experimenter upon the scalp in order to stimulate the prefrontal cortex (PFC) bilaterally.

#### rTMS Protocol

After the recruitment phase, the subjects were randomly assigned to active (10 Hz) or sham stimulation protocol in a double-blind fashion. All subjects received 3 stimulations per week along 4 weeks, for a total amount of 12 stimulations. The intensity of stimulation was set at 100% of individual resting motor threshold, defined as the lowest intensity needed to obtain 5/10 evoked motor potentials (MEP) greater than 50 μV from the abductor pollicis brevis (APB) of the right hand. We stimulated PFC bilaterally at 10 Hz frequency that is considered able to enhance the neural response (13, 38). Each stimulation included 20 trains of 50 pulses each with 15 s of inter-stimulus between trains. Each train duration was 5 s long (10 pulses/s) for a total amount of 1000 pulses. The sham protocol was settled with the same parameters without delivering any pulses. Each session lasted about 10 min.

### Statistics

Multiple independent samples Student’s *t*-test was used to compare demographic features, duration of dependence, and toxicological data between the two experimental groups at baseline. Two-way and one-way Analysis of Variance (ANOVA) for repeated measures was used to evaluate the effects of rTMS on cocaine intake between and within groups over the time line considered. We therefore applied a *post hoc* comparison analysis between T0 and each subsequent time points (T1, T2, and T3). *p* < 0.05 was considered statistically significant. We normalized the toxicological data in order to have a common baseline starting from the unit (“1”) to allow easy comparisons between the two groups.

## Results

### Participants

We recruited 18 subjects affected by CUD (DSM-V), 16 males, and 2 females matched for age, education, and duration of addiction (Table 1).

**Table 1:** **Demographic characteristics at baseline (M ± SD and ranges)**.

	rTMS group (*n* = 10)	Control group (*n* = 8)
Gender (M/F)	9/1	7/1
Age	33.9 ± 6.5 (27–48)	32.4 ± 10.6 (23–50)
Education (years)	11 ± 3.5 (8–13)	11.75 ± 2.31 (8–13)
Employed (yes/no)	7/3	4/4
Duration of cocaine dependence (years)	12.3 ± 5.7	9.1 ± 6.1
Cocaine amount in the hair (ng/mg)	11.31	8.77

We did not observe any discomfort in the subjects exposed to the treatment except for a patient who suffered from a mild headache after receiving active stimulation. Three subjects from the sham group (37%) and one subject from the active group (10%) abandoned the study before ending the 12 sessions of stimulation (four dropouts). Four more subjects were excluded because of the extremely high amount of cocaine measured at T0 that was abundantly out of accepted range (four outliers). Two subjects out of four (50%) from the sham group relapsed three times after the treatment, while two subjects out of six (33%) from the active group reported one relapse after 6 months from the treatment. At the end of the study, 10 cocaine addicts were admitted to the statistics analysis, 6 patients from the active group [1 female, 5 males; average age 35 years; average duration of cocaine dependency 13 years; average cocaine intake (T0) 7.75 ng/mg], and 4 patients from the sham group [1 female; 3 males; average age 34.5 years; average duration of cocaine dependency 11.75 years; average cocaine intake (T0) 6.53 ng/mg].

### Toxicological Data

**Figure 1:**
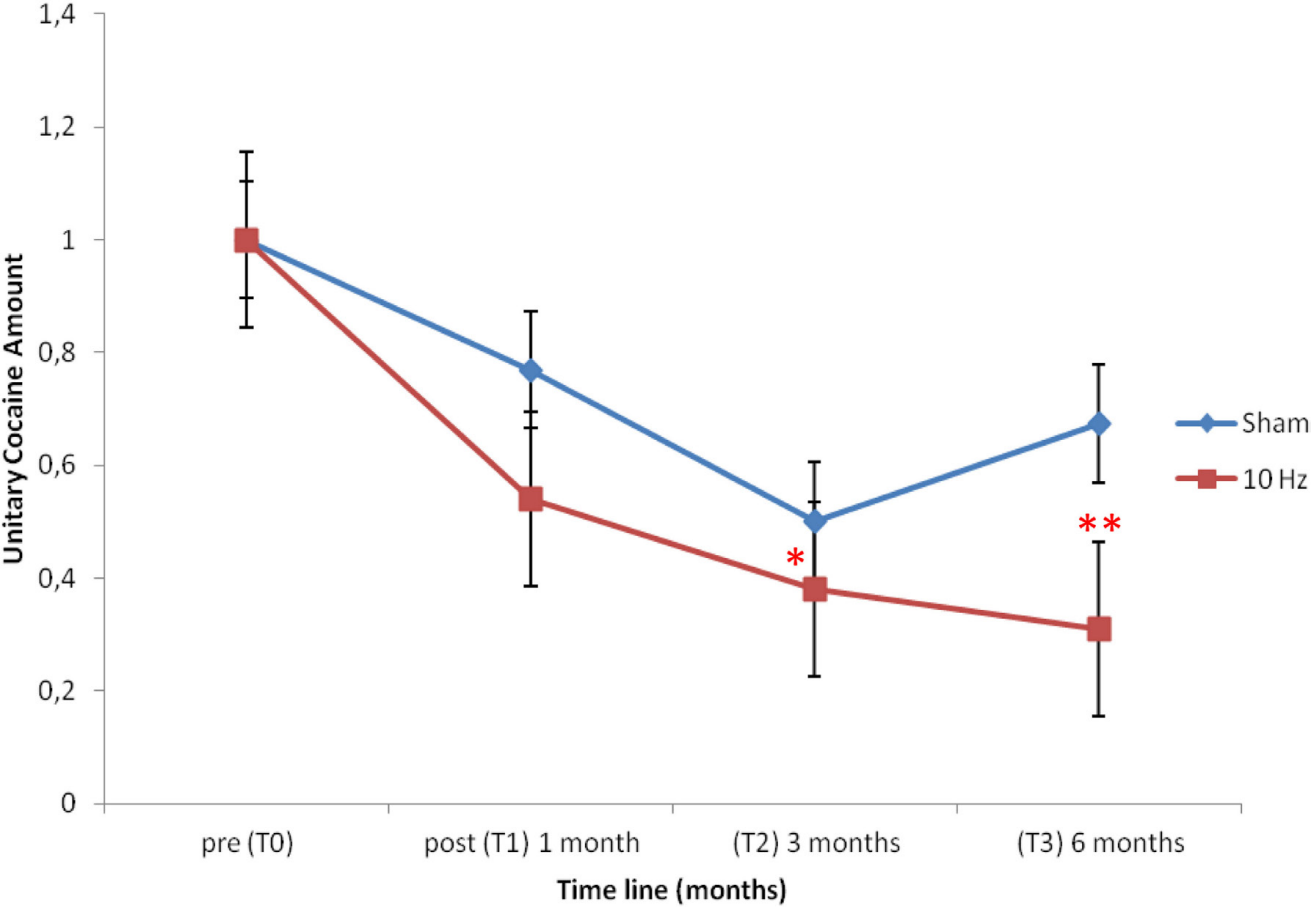
**Depicts the unitary cocaine amount in the two experimental groups along the time line considered from the baseline (T0) till 6 months later (T3)**. Note that only in the active group (10 Hz) there is a significant reduction in the unitary cocaine amount as indexed by T0–T2 (*p = 0.02) and T0–T3 (**p = 0.01) comparison over the time line.

All subjects resulted positive for cocaine and negative for opioid and cannabis at the hair test analysis at the baseline (T0). The hair samples were collected for each group until 6 months after the treatment. The two-way ANOVA for repeated measures was performed in order to highlight differences between the active stimulation effect and the sham over time; no significant effect resulted in the interaction between time and treatment (*F*_4,32_ = 0.35; *p* = 0.84), which indicated no difference in the effect of the two experimental condition (active vs. sham) on cocaine intake (Table 2). However considering the paucity of the studies available, we performed exploratory analysis, which indicated a significant reduction in cocaine intake between T0 and T2 (*t* = 3.30; *p* = 0.02) and T0–T3 (*t* = 3.72; *p* = 0.01) time in the active TMS group as highlighted by Bonferroni correction (Figure 1). Moreover, the one-way ANOVA for repeated measures with time as factor conducted to explore in depth the toxicological data within groups showed a significant reduction on cocaine intake in the active group (*F*_3,23_ = 3.42; *p* = 0.04) and not in the sham (*F*_3,15_ = 1.88; *p* = 0.20). *Post hoc* comparison (Duncan Test) showed a significant difference in cocaine amount at T0 compared to T3 (*p* < 0.05) in the active group.

**Table 2:** **Repeated measure analysis of variance (two-way ANOVA)**.

Two-way ANOVA Alpha <0.05	SS	DF	MS	*F*	*p* value
Interaction	0.2462	4	0.06	*F*_4,32_ = 0.35	0.84
Time	3.87	4	0.97	*F*_4,32_ = 5.54	0.001**
Column factor	0.33	1	0.33	*F*_1,8_ = 0.29	0.60
Subjects (matching)	9.04	8	1.13	*F*_8,32_ = 6.46	<0.00
Residual	5.59	32	0.17		

The table shows the main effect of “Time” (**p = 0.001). The *post hoc* comparison reveals a consisting reduction in the cocaine amount only in the active group as demonstrates by the T0–T2 (*p* = 0.02) and T0–T3 (*p* = 0.01) index.

## Discussion

In the present study, we used high-frequency stimulation (10 Hz) with the double objective to hasten neural activity in the bilateral PFC, which is known to be hypoactive in addicts (39), and to facilitate output toward subcortical areas and provide an activation of dopaminergic transmission, known to be hypofunctional in cocaine addicts as indexed by previous basic and human studies (9). However, in contrast to previous studies, we tested the effects of TMS on cocaine intake both in active and sham control group in a double-blind manner to minimize potential bias, over a 6-month long observation. Our data did not show a significant effect of the interaction between treatment and time, which indicated that the two interventions did not differ in the effects on cocaine intake, as shown by the two-way ANOVA. However, the paucity of the sample can play a role in the result of the statistical analysis and it is not improbable that the enlargement of the sample would turn the decreasing trend in cocaine intake observed in the active group vs. sham into a statistical significance. Indeed, when we performed exploratory analysis, we found a long-lasting decreasing on cocaine intake only in the active group (T0–T2; T0–T3) and not in the sham. The significant reduction in cocaine intake along time observed in the active group is not associated to a significant short-term difference (T1–T2), likely because of the intrinsic characteristics of the hair analysis. Indeed, this test is used to determine the history and severity of an individual’s drug use, and it has been shown to become negative at least 3 months after the last intake. The rationale of its utilization in this study is based on the evidence that it provides long-term information about cocaine assumption, with higher sensitivity and specificity, and in a wider surveillance window than urine test (40–42). The possibility of a “false negative” effect played by the limited number of patients seems to be unlikely, although it cannot be completely ruled out. Notably, a lower rate of dropouts was observed in the active group (10%) compared to sham (37%): this could be due to the modulation of specific circuitries related to motivation cognition and compliance by the active TMS that strengthens the patients’ adherence to the treatment.

In conclusion, the present data are in line with previous studies that support a beneficial role for rTMS in CUDs (20–22) and encourage the investigation on the role of the rTMS parameters in modulating the efficacy of stimulation that are matter of intense debate (43). Indeed, the frequency/pattern of stimulation seems to be a key element in the effects observed. A recent report (20) employed 15 Hz twice a day for five consecutive days and obtained a reduction in cocaine intake. Likewise, Ceccanti et al. (18) reported a reduction in alcohol intake after 10 sessions of 20 Hz TMS in alcoholics. These results prompt clinicians and researchers to further investigate on stimulation frequency/pattern as the key element to obtain lasting effects that exploit the principles of neural plasticity (43). Another important issue to comment is on the geometry of the coil employed: the common figure-of-eight coil (20, 21) delivers a focal unilateral (asymmetric) stimulation site whereas the novel design of H1-coil (37) delivers bilateral simultaneous stimuli that can modulate prefrontal cortical areas that project into subcortical pathways. Since addiction is whole brain pathology with no evidence of lateralization (44, 45), the H-coil may represent a more suitable tool for future clinical studies aimed at reducing drug intake, especially taking advantage from the ultimate H4-coil. Indeed, bilateral simultaneous TMS application can be more efficacious in yielding a robust and bilateral dopaminergic “boost” as it has been shown in visual imaging studies (46).

Our results, although preliminary and limited by the very small sample available, indicate that bilateral rTMS of PFC may induce a long-lasting reduction in cocaine intake in subjects diagnosed with CUD according to DSM-V criteria. Future studies will be aimed at optimizing both the sample and the protocol characteristics, in order to contribute to the overall definition of the rTMS as a valid tool for the treatment of addiction.

## Author Contributions

CB and RP delivered TMS treatment. CB, AF, and MP performed clinical evaluation. CG performed hair analysis. TP, GM, AB, and CC performed statistical analysis of the data. CB and MD wrote the manuscript. MD conceived experiments and provided funding.

## Conflict of Interest Statement

The authors declare that the research was conducted in the absence of any commercial or financial relationships that could be construed as a potential conflict of interest.
