# Supplementary material for: Bilateral Transcranial Magnetic Stimulation of the Prefrontal Cortex Reduces Cocaine Intake: A Pilot Study
Source: Front Psychiatry. 2016 Aug 8;7:133. doi: 10.3389/fpsyt.2016.00133 (PMC4976094; doi:10.3389/fpsyt.2016.00133)
Supplement: Supplementary file 1 [file data_sheet_1.doc]

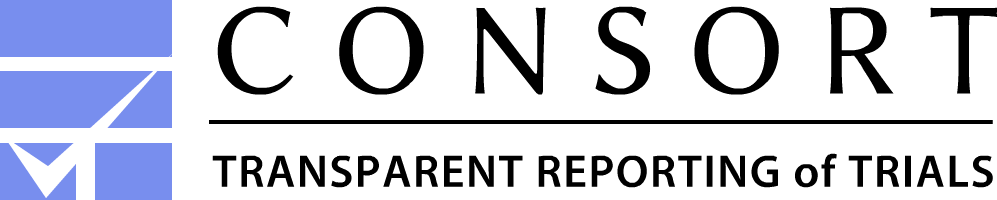


**CONSORT 2010 Flow Diagram**

**Allocation**

**Analysis**

**Follow-Up**

**Enrollment**

Assessed for eligibility (n=18)

Excluded (n=0)

♦  Not meeting inclusion criteria (n=0)

♦  Declined to participate (n=0)

♦  Other reasons (n=0)

Analysed (n=6)
♦ Excluded from analysis (outlier) (n=3)

Lost to follow-up (give reasons) (n=0)

Discontinued intervention (give reasons) (n=0)

Allocated to intervention (n=10)

♦ Received allocated intervention (n=9)

♦ Did not receive allocated intervention (drop-out) (n=1)

Lost to follow-up (give reasons) (n=0)

Discontinued intervention (give reasons) (n=0)

Allocated to intervention (n=8)

♦ Received allocated intervention (n=5)

♦ Did not receive allocated intervention (drop-out) (n=3)

Analysed (n=4)
♦ Excluded from analysis (outlier) (n=1)

Randomized (n=18)
