# Supplementary figures and images for: Bilateral Transcranial Magnetic Stimulation of the Prefrontal Cortex Reduces Cocaine Intake: A Pilot Study
Source: Front Psychiatry. 2016 Aug 8;7:133. doi: 10.3389/fpsyt.2016.00133 (PMC4976094; doi:10.3389/fpsyt.2016.00133)

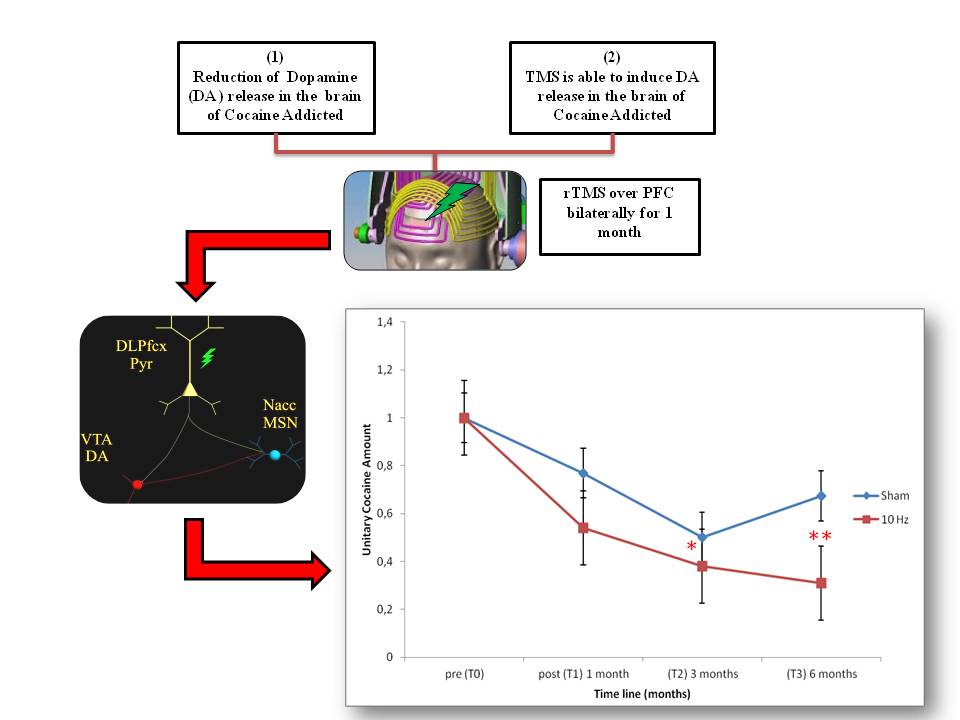

Supplement: Supplementary file 3 [file image_1.jpeg]
